# Therapeutic properties of multi-cannabinoid treatment strategies for Alzheimer’s disease

**DOI:** 10.3389/fnins.2022.962922

**Published:** 2022-09-02

**Authors:** Madilyn Coles, Genevieve Z. Steiner-Lim, Tim Karl

**Affiliations:** ^1^School of Medicine, Western Sydney University, Campbelltown, NSW, Australia; ^2^NICM Health Research Institute and Translational Health Research Institute (THRI), Western Sydney University, Penrith, NSW, Australia; ^3^Neuroscience Research Australia, Randwick, NSW, Australia

**Keywords:** dementia, Alzheimer’s disease, cannabis extract, cannabidiol (CBD), delta-9-tetrahydrocannabinol (THC), cannabis therapeutics, endocannabinod system

## Abstract

Alzheimer’s disease (AD) is a debilitating neurodegenerative disease characterized by declining cognition and behavioral impairment, and hallmarked by extracellular amyloid-β plaques, intracellular neurofibrillary tangles (NFT), oxidative stress, neuroinflammation, and neurodegeneration. There is currently no cure for AD and approved treatments do not halt or slow disease progression, highlighting the need for novel therapeutic strategies. Importantly, the endocannabinoid system (ECS) is affected in AD. Phytocannabinoids, including cannabidiol (CBD) and Δ^9^-tetrahydrocannabinol (THC), interact with the ECS, have anti-inflammatory, antioxidant, and neuroprotective properties, can ameliorate amyloid-β and NFT-related pathologies, and promote neurogenesis. Thus, in recent years, purified CBD and THC have been evaluated for their therapeutic potential. CBD reversed and prevented the development of cognitive deficits in AD rodent models, and low-dose THC improved cognition in aging mice. Importantly, CBD, THC, and other phytochemicals present in *Cannabis sativa* interact with each other in a synergistic fashion (the “entourage effect”) and have greater therapeutic potential when administered together, rather than individually. Thus, treatment of AD using a multi-cannabinoid strategy (such as whole plant cannabis extracts or particular CBD:THC combinations) may be more efficacious compared to cannabinoid isolate treatment strategies. Here, we review the current evidence for the validity of using multi-cannabinoid formulations for AD therapy. We discuss that such treatment strategies appear valid for AD therapy but further investigations, particularly clinical studies, are required to determine optimal dose and ratio of cannabinoids for superior effectiveness and limiting potential side effects. Furthermore, it is pertinent that future *in vivo* and clinical investigations consider sex effects.

## Alzheimer’s disease

Alzheimer’s disease (AD) is a disabling neurodegenerative disease and the most common form of dementia. Dementia due to AD is clinically characterized by cognitive decline (e.g., language, memory, and executive function) and functional impairment in activities of daily living (Alzheimer’s Association, 2021). AD is caused by irreversible and progressive neuronal dysfunction and cell death, which causes cerebral atrophy, and is pathologically hallmarked by the extracellular accumulation of aberrant amyloid-β (Aβ; particularly Aβ_42_) peptides into plaques, hyperphosphorylation of microtubule associated protein tau (MAPT, or simply tau) leading to the formation of neurofibrillary tangles (NFTs), and neuroinflammation (Cai et al., 2014; Heneka et al., 2015; Alzheimer’s Association, 2021). Together, this pathophysiology causes mitochondrial dysfunction, an antioxidant system/redox imbalance, and increased reactive oxygen species (ROS), which further promotes aggregation of Aβ and tau hyperphosphorylation, creating a vicious cycle (Zhao and Zhao, 2013). Neuroinflammation and Aβ pathology also cause glutamatergic dysfunction (Wang and Reddy, 2017) creating an excito-neurotoxic state and neurodegeneration (Parsons et al., 2013) affecting cholinergic neurons, decreasing acetylcholine (ACh) (Schliebs and Arendt, 2011). Finally, hippocampal neurogenesis is attenuated in AD and contributes further to memory impairment (Mu and Gage, 2011; Babcock et al., 2021).

Signaling impairments within the glutamatergic and cholinergic systems are the main targets of currently approved AD therapies (Parsons et al., 2013). However, the therapeutic efficacy of acetylcholinesterase inhibitors (rivastigmine, donepezil, and galantamine) and NMDA receptor antagonist (memantine) is only modest, cause numerous side effects, and do not modify disease progression (Wong, 2016). Further, the recent FDA approval of the monoclonal antibody agent, aducanumab, has been controversial given its failure to demonstrate clinical efficacy despite reducing Aβ (Knopman et al., 2021). Thus, novel therapeutic advancements for AD beyond Aβ and neurotransmitter imbalance are needed.

## Changes to the endocannabinoid system in Alzheimer’s disease

In recent years, the therapeutic value of targeting the endocannabinoid system (ECS) and evaluation of phytocannabinoids as treatment options for AD have become major focus points in the field. The ECS is composed of cannabinoid receptors 1 and 2 (CB1R and CB2R, encoded by *CNR1* and *CNR2*), endocannabinoids [including anandamide (AEA) and 2-aracidonoylglycerol (2-AG)], and enzymes involved in the biosynthesis or degradation of endocannabinoids (Aizpurua-Olaizola et al., 2017). Physiologically, the ECS is involved in the homeostasis of numerous functions of the human body including cognition (e.g., learning, memory), anxiety, neurogenesis, pain sensation, immune signaling, and inflammation (Baker et al., 2003; Sinclair, 2020). Alterations to the ECS have been found in AD, with conflicting views that endocannabinoid signaling is upregulated to counteract neuronal hyperactivity and neuroinflammation, or that the upregulation itself contributes to AD symptoms such as memory loss (Di Marzo et al., 2004).

CB2R upregulation has been consistently found *ex vivo* in the brain tissue of people with AD and AD-relevant rodent models, with evidence suggesting that CB2R expression is activated due to immunomodulation induced by the pathogenic events present in AD. For example, in two clinical studies, upregulated CB2R was found in microglia proximal to plaque-associated hippocampal tissue (Benito et al., 2003) and positively correlated with Aβ_42_ levels and plaque deposition (Solas et al., 2013). Similarly, increased levels of CB2R and 2-AG have been detected in Aβ_42_-treated rats (Esposito et al., 2007a). Furthermore, stimulation of microglial CB2R induced *in situ* removal of native Aβ from human AD tissue sections (Tolón et al., 2009), and *in vitro* and *in vivo* stimulation of CB2R blocked Aβ-induced activation of microglia (Ramírez et al., 2005). Chronic CB2R agonism also resulted in cognitive improvement and reduced microglial reactivity in *APP_Swe_/PS1*Δ*E9* mice (B6; C3-Tg[APPswe,PSEN1dE9]85Dbo/Mmjax or *APP/PS1* mice; a double transgenic mouse model of AD with disease-relevant mutations in *amyloid precursor protein* [*APP*] and *presenilin 1* [*PS1*]). Interestingly, this beneficial effect was without associated improvement of amyloid burden (Aso et al., 2013). Further, *APP/PS1*/CB2R^–/–^ mice (*APP/PS1* mice with additional *CNR2* knockout) exhibited reduced levels of microglia, infiltrating macrophages, pro-inflammatory chemokines and cytokines, reduced soluble Aβ (Schmöle et al., 2015), and exacerbated cortical Aβ deposition (Aso et al., 2016a).

Conversely, reductions in CB1R have been detected in areas of microglial activation in brains of both people with AD and AD-relevant rodent models (Ramírez et al., 2005), and decreased levels of CB1R and AEA have also been found in Aβ_42_-treated rats (Esposito et al., 2007a). Conversely, 3xTg-AD mice exhibited upregulated CB1R in the prefrontal cortex, amygdala, and dorsal hippocampus, but downregulated CB1R in the ventral hippocampus (Bedse et al., 2014). In addition, *APP/PS1*/CB1R^–/–^ mice (*APP/PS1* mice with additional *CNR1* knockout) developed accelerated memory impairments in the two-object recognition test (Aso et al., 2018). Finally, downregulated AEA in cortical AD post-mortem brain tissue was inversely correlated with Aβ_42_ levels and cognitive symptoms (Jung et al., 2012). In summary, there is clear evidence for a role of the ECS in AD, which makes the ECS a desirable therapeutic target, although the AD-ECS relationship appears to be complex and requires further investigation.

## Effects of phytocannabinoids on Alzheimer’s disease

**FIGURE 1:**
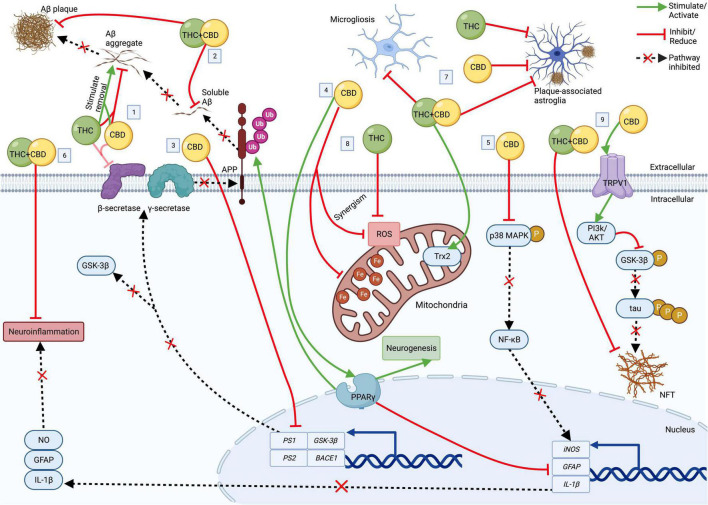
The cellular effects of CBD, THC, and CBD+THC in AD. Top: Extracellular; Bottom: Intracellular. **(1)** CBD and THC each block Aβ deposition and ↑ aggregated Aβ removal. CBD and THC also (weakly) inhibit β-secretase. **(2)** CBD+THC ↓ Aβ plaques and soluble Aβ_42_. **(3)** CBD ↓ transcription of *PS1*, *PS2*, *BACE1*, and *GSK-3*β, resulting in ↓ enzymes involved Aβ and tau production. **(4)** Acting *via* PPARγ, CBD (a) induces APP ubiquitination, resulting in ↓Aβ, (b) ↑ neurogenesis, and (c) ↓ neuroinflammation by suppressing *GFAP*, *IL-1*β and *iNOS* expression. **(5)** CBD also ↓ *iNOS via* p38 MAPK and NF- κB. **(6)** CBD+THC also ↓ neuroinflammation. **(7)** CBD, THC, and/or CBD+THC ↓astrogliosis and microgliosis. CBD+THC also ↑ Trx2. **(8)** CBD and THC ↓ROS (with some potential synergism). CBD also ↓ mitochondrial ferritin. **(9)** CBD ↓ tau hyperphosphorylation *via* a TRPV1/PI3K/AKT/GSK-3β axis. CBD+THC also ↓ NFT. Aβ, Amyloid-β; APP, amyloid precursor protein; AD, Alzheimer’s disease; BACE1, β-secretase 1; CBD, cannabidiol; Fe, ferritin; GFAP, glial fibrillary acidic protein; GSK-3β, glycogen synthase kinase 3β; iNOS, inducible nitric oxide synthase; IL-1β, interleukin 1 beta; NFT, neurofibrillary tangle; NO, nitric oxide; NF-κB, nuclear factor-κB; p38 MAPK, p38 mitogen-activated protein kinase; P, phosphate group; PI3K/Akt, phosphatidylinositol 3-kinase/Akt kinase; PPARγ, peroxisome proliferator-activated receptor gamma; PS1, presenilin 1; PS2, presenilin 2; ROS, reactive oxygen species; THC, delta-9-tetrahydrocannabinol; TRPVI, transient receptor potential vallinoid 1; Trx2, thioredoxin 2; Ub, ubiquitin. Created with BioRender.com.

Phytocannabinoids, including cannabidiol (CBD) and Δ9-tetrahydrocannabinol (THC) have gained attention as a potential therapeutic strategy for dementia including AD. CBD (non-intoxicating) and THC modulate the ECS, are neuroprotective, anti-inflammatory, and antioxidant, and emerging evidence suggests that they have therapeutic-like effects on Aβ accumulation and tau hyperphosphorylation (reviewed in Karl et al., 2017). In the following, we will highlight the therapeutic properties of CBD, THC, other minor cannabinoids and cannabinoid acids, and multi-cannabinoid treatment strategies for AD (for details on the pharmacological profile of CBD and THC; see Grotenhermen, 2003; Pertwee, 2008). Figure 1 summarizes the extracellular and intracellular effects of CBD, THC, and CBD combined with THC (CBD+THC) in AD.

*In vitro*, CBD possesses several characteristics directly relevant to therapeutic efficacy in AD. CBD inhibited tau hyperphosphorylation in Aβ-treated rat PC12 neuronal cells *via* reduced glycogen synthase kinase 3β (GSK-3β; responsible for tau hyperphosphorylation) (Esposito et al., 2006a), which was later shown to be *via* down-regulation of *GSK*-3β transcription as well as *via* activation of transient receptor potential vallinoid 1 (TRPVI) and subsequent promotion of phosphatidylinositol 3-kinase/Akt kinase (PI3K/Akt) signaling in mesenchymal stem cells (Libro et al., 2016). CBD also increased cell survival, reduced lipid peroxidation and ROS production (Iuvone et al., 2004) and attenuated nitric oxide production [*via* inhibition of phosphorylated p38 mitogen-activated protein kinase (p38 MAPK) and transcription factor nuclear factor-κB (NF- κB)] in Aβ-treated rat PC12 neuronal cells (Esposito et al., 2006b). Importantly, CBD also decreased APP expression in APP-transfected human neuroblastoma cells by inducing APP ubiquitination through peroxisome proliferator-activated receptor gamma (PPARγ), which was paralleled by a reduction of Aβ peptide expression and increased cell survival (Scuderi et al., 2014). Additionally, CBD down-regulated the *β-secretase 1* (*BACE1*), *PS1*, and *presenilin 2* (*PS2*) genes (Libro et al., 2016) that code for the β- and γ-secretases involved in the enzymatic generation of Aβ. Interestingly, CBD has also been found to directly inhibit β-secretase (although weakly) (Mooko et al., 2021). Furthermore, CBD was protective against amyloid toxicity in an inducible human neuron-like cell model of AD (MC65 cells) (Schubert et al., 2019), and hemp seed oil (80% CBD) was 25% effective in reducing Aβ_42_+Cu (II)-induced ROS in SH-SY5Y cells (Raja et al., 2020). CBD has also shown potential to reverse mitochondrial dysfunction, a core feature of AD. For example, in an *in vivo* brain iron overload model (iron-treated rats), CBD rescued iron-induced apoptosis, restored levels of hippocampal dynamin-related protein 1 (a mitochondrial fission protein), and reversed increased mitochondrial ferritin and altered mitochondrial epigenetic modulation, leading to increased neuronal survival (da Silva et al., 2014, 2018a,b). Additionally, CBD has been found to reduce oxidative stress *via* attenuation of ROS and ROS-generating reduced nicotinamide adenine dinucleotide phosphate (NADPH) oxidase (NOX) isoforms (Vallée et al., 2017). See Figure 1 for several of the mentioned effects of CBD on pathologies in AD.

*In vivo*, 7 days of CBD treatment dose-dependently attenuated Aβ-evoked neuroinflammation [e.g., CBD inhibited expression of *glial fibrillary acidic protein* (*GFAP*), *inducible nitric oxide synthase* (*iNOS*), and *interleukin 1 beta* (*IL-1*β)] in a pharmacological mouse model of AD (Esposito et al., 2007b), which was mediated *via* PPARγ and associated with increased neurogenesis (see Figure 1; Esposito et al., 2011). Chronic treatment with 20 mg/kg CBD also prevented learning deficits in an Aβ pharmacological mouse model (Martín-Moreno et al., 2011). In *APP/PS1* transgenic mice, chronic treatment with various CBD doses (5–50 mg/kg) reversed and (at 20 mg/kg) prevented the development of several cognitive deficits (Cheng et al., 2014a,b; Coles et al., 2020; Watt et al., 2020b). Interestingly, CBD only moderately reduced insoluble Aβ_40_ and pro-inflammatory cytokines and had no other effects on AD brain pathology. In a tauopathy model of AD (i.e., TAU58/2 transgenic mice), CBD improved spatial memory of 14-month-old female mice (but not of 4-month-old males). CBD also did not reverse motor impairments of 4- or 14-month-old AD transgenic animals (Watt et al., 2020a; Kreilaus et al., 2022). To date, no clinical trials have been published regarding CBD therapy for dementia or AD, however, several trials are underway according to international clinical trial registries (e.g., NCT04436081, 2019-002106-52, ACTRN12621001364864).

THC has also been found to inhibit several AD-related pathologies. For example, *in vitro* studies showed that THC dose-dependently removed and inhibited the aggregation of Aβ in neuro2a Swedish variant APP cells (Cao et al., 2014) and in induced MC65 cells (Currais et al., 2016; Schubert et al., 2019) and protected against excitotoxicity and oxidative stress in mouse neuronal cells (Marsicano et al., 2002). Furthermore, 80% THC cannabis extract was 60% effective in reducing Aβ_42_+Cu (II)-induced ROS in SH-SY5Y cells (Raja et al., 2020). Additionally, THC weakly inhibits β-secretase (Mooko et al., 2021; see Figure 1). Finally, *in silico* modeling demonstrates that THC prevents Aβ aggregation *via* acetylcholinesterase inhibition (Eubanks et al., 2006).

*In vivo* studies show that low-dose THC can improve cognitive performance. For example, (sub) chronic administration of 1.5 mg/kg THC improved the learning and memory of male Sprague Dawley rats (Suliman et al., 2018), and a single injection of 0.002 mg/kg THC reversed age-associated cognitive impairments in 24-month-old female ICR mice (Sarne et al., 2018). Importantly, chronic administration of 1 and 3 mg/kg THC restored cognitive function of 12- (only 3 mg evaluated) and 18-month-old male C57BL/6J mice (Bilkei-Gorzo et al., 2017; Nidadavolu et al., 2021). However, low-dose THC treatment of 2-month-old C57BL/6J male mice negatively impacted spatial memory performance, suggesting that THC-only treatments are not an ideal treatment strategy for improving cognition in AD (Bilkei-Gorzo et al., 2017).

In people with all-cause dementia or AD, THC has been shown to improve weight gain and non-cognitive symptoms [behavioral and psychological symptoms of dementia (BPSD); e.g., agitation] (Volicer et al., 1997; Walther et al., 2006, 2011; Herrmann et al., 2019; Charernboon et al., 2021). Other studies have found no beneficial effects of THC for dementia, but conclude that up to 4.5 mg THC daily is well tolerated (van den Elsen et al., 2015a,b), while others report some negative side effects including somnolence (Volicer et al., 1997). Two further trials investigating THC for treatment of agitation in AD are underway (e.g., NCT04516057, NCT02792257).

The therapeutic benefits of THC for AD may be impeded by adverse effects including (but not limited to) psychoactivity, dizziness, disorientation, and anxiety (Whiting et al., 2015), particularly relating to high-dose THC administration (e.g., > 10 mg orally for humans, or > 10 mg/kg THC i.p. for mice) (Paronis et al., 2012; Calabrese and Rubio-Casillas, 2018). Importantly, the side effect profile of THC may lessen over time due to tolerance effects (Haney et al., 1999) or may be offset or reduced by using a low-dose regime (e.g., up to ∼3 mg/day orally for humans or < 3 mg/kg i.p. for mice). Furthermore, inclusion of other cannabinoids, particularly CBD, into the formulation may help to block the negative effects of THC (due to the “entourage effect”; see section “Treatment effects of cannabinoid combinations and cannabis extracts in Alzheimer’s disease”). Given this, and that low-dose THC has potential to improve cognition *in vivo* and improve BPSD in clinical trials (albeit weak evidence), inclusion of low levels of THC in a cannabis-based AD medication maybe be of value, despite the side effect profile at higher doses.

Cannabinol (CBN), cannabigerol (CBG), cannabichromene (CBC), cannabidivarin (CBDV), and the THC and CBD acids (THCA and CBDA) are minor anti-inflammatory phytocannabinoids that also have neuroprotective properties for AD and could be used as alternatives to THC as they are largely non-intoxicating (or in the case of CBN, less psychoactive than THC) (Steiner-Lim et al., under review). Briefly, Schubert et al. (2019) found that CBN, CBC, CBDV, and CBDA prevented amyloid toxicity/cell death following removal of tetracycline from MC65 cells. Furthermore, CBN, CBG, CBC, and CBDV were found to not only block the accumulation of Aβ, but they also stimulated the degradation and removal of preformed Aβ aggregates. In addition, CBN, CBG, CBC, CBDV, and THCA prevented oxytosis, which in the case of CBN, was later confirmed to be *via* direct targeting of mitochondria and promotion of antioxidant defenses, indirectly of CB receptors (Liang et al., 2022). CBC has also been suggested to have pro-neurogenic benefits *via* suppression of reactive astrocytes (Shinjyo and Di Marzo, 2013), and THCA has demonstrated numerous PPARγ-dependent neuroprotective properties both *in vitro* and *in vivo* (Nadal et al., 2017).

## Treatment effects of cannabinoid combinations and cannabis extracts in Alzheimer’s disease

Research suggests that cannabinoid treatments involving a combination of THC and CBD and other cannabinoids can produce greater therapeutic outcomes and less adverse effects than treatment with purified cannabinoid isolates. For example, CBD can oppose the undesirable effects of THC (e.g., intoxication and sedation), while potentiating the analgesic and anti-emetic properties (as reviewed in Russo and Guy, 2006). This is thought to be due to the “entourage effect” which refers to the tendency of cannabinoids to interact when administered together, but also the idea that the terpenoids and flavonoids present in cannabis extracts may contribute to the overall synergy of the treatment (Russo, 2011). Importantly, and in line with the knowledge that cannabinoids work in a dose-dependent and biphasic manner (Tzavara et al., 2003; Rey et al., 2012), the dosage and ratios at which cannabinoid combinations are administered affect the overall therapeutic profile (Zuardi et al., 2012). For example, it has been suggested that a 8.1:1 CBD:THC ratio might result in the observation of antagonism of CBD on THC-induced effects while a ratio of 1.8:1 CBD:THC might result in potentiation of THC effects (Zuardi et al., 2012). The following section will discuss the evidence currently available for the validity of multi-cannabinoid treatment strategies in AD therapy, with a focus on CBD and THC, given the infancy of research involving other minor cannabinoids discussed above (summarized in Table 1).

**TABLE 1:** Summary of the effects of CBD:THC cannabinoid combination treatments in AD-relevant cell and animal models as well as in people with AD from clinical studies.

Model System	Treatment	Effect	References
***In vitro* studies**			
Oxytosis induced HT22	THC+CBD	Additive neuroprotective effect against ROS	Schubert et al., 2019
cells (*via* glutamate)	THC+CBN	Synergistic neuroprotective effect against ROS, non-ECS mechanism	
Oxytosis induced SH-SY5Y cells (*via* H_2_O_2_)	THC, CBD	THC (IC_50_ = 0.4 μg/mL) had a higher potency in combating ROS *cf.* CBD (IC_50_ = 42.7 μg/mL)	Raja et al., 2020
	THC-rich cannabis extracts	↓ ROS by 70–80%, IC_50_ = 0.4–1.2 μg/mL	
	CBD-rich cannabis extracts	↓ ROS by 60+%, IC_50_ = 0.5–0.6 μg/mL	
	Combinations of CBD:THC	10:90 CBD:THC; IC_50_ = 2.5 μg/mL 25:75 CBD:THC; IC_50_ = 0.4 μg/mL, most effective antioxidant 50:50 CBD:THC, IC_50_ = 0.5 μg/mL 75:25 CBD:THC, IC_50_ = 1 μg/mL 90:10 CBD:THC, IC_50_ = 5 μg/mL, least effective antioxidant	
***In vivo* studies**			
Young AD model *APP/PS1* transgenic male mice 6 months old	CBD-rich cannabis extract (0.75 mg/kg CBD), daily i.p., 5 weeks	Reversed the object recognition memory deficit ↓ astrogliosis	Aso et al., 2015
	THC-rich cannabis extract (0.75 mg/kg THC), daily i.p., 5 weeks	Reversed the object recognition memory deficit ↓ astrogliosis ↓ the object recognition memory of WT mice***	
	1:1 CBD:THC cannabis extract (0.75 mg/kg each CBD and THC), daily i.p., 5 weeks	Reversed the object recognition memory deficit and improved learning impairments ↓ cortical soluble Aβ_42_ peptides (and ↑ Aβ_42_/ Aβ_42_ ratio in amyloid plaques) ↓ astrogliosis, microgliosis, and modified inflammatory markers No effect on the object recognition memory of WT mice	
Aged AD model *APP/PS1* transgenic male mice 12 months old	1:1 CBD:THC cannabis extract (0.75 mg/kg each CBD and THC), daily i.p., 5 weeks	Reversed the object recognition memory deficit No effect on amyloid pathology or gliosis No effect on the age-related object recognition memory deficit of aged WT mice *cf.* non-aged controls	Aso et al., 2016b
Tauopathy model PK^–/–^/Tau^VLW^ transgenic male mice 6 months old	1:1 CBD:THC cannabis extract (1.5 mg/kg each CBD and THC), daily i.p., 1 month	↓ hippocampal and cerebral Aβ and tau deposition and ↑ autophagy ↓ neuroinflammation and gliosis ↑ reduced/oxidized glutathione ratio, ↓ levels of free radicals and iNOS ↓ stress, aggressive behavior, and stereotypy	Casarejos et al., 2013
Aged mice C57BL/6J male mice 18 months old	THC (1 mg/kg), daily s.c. *via* osmotic mini pump, 4 weeks	↑ spatial learning No effect on spatial memory	Nidadavolu et al., 2021
	1:1 CBD:THC (1 mg/kg each CBD and THC), daily s.c. *via* osmotic mini pump, 4 weeks	No effect on spatial learning or memory	
Lafora disease model Mice homozygous for the *EPM2B* deletion (malin knock-out; sex not specified) 4 and 10 months old	CBD-rich cannabis extract (35 mg/kg CBD, 4.8 mg/kg THC), 5 days per week p.o., 2 months	Reversed the object recognition memory deficit of 12-month-old malin knock-out mice ↓ the object recognition memory of 6-month-old WT mice***	Aso et al., 2020
**Clinical studies**			
10 people with AD and BPSD, open label prospective trial gender not specified	THC-rich cannabis extract (2.5 mg THC, titrated to 5 or 7.5 mg in 3 patients), two times daily p.o., 4 weeks, adjunctive therapy to usual care	↓ Clinical Global Impression severity scale from 6.5 to 5.7 ↓ Neuropsychiatric Inventory scale from 44.4 to 12.8; improvements to delusions, agitation/aggression, irritability, apathy, sleep, and caregiver distress Side effects: confusion in one patient at 5 mg***	Shelef et al., 2016^
10 women with severe dementia and BPSD, prospective observational pilot study	CBD:THC tincture or oil (average 13.2–18 mg/day CBD and 7.6–9 mg/day THC, titrated), three times daily p.o., 2 months	↓ Neuropsychiatric Inventory scale from 71.1 to 38.3 ↓ agitation (Cohen-Mansfield Agitation Inventory) from 74.5 to 47.5 ↓ rigidity (Unified Parkinson’s Disease Rating scale) from 3.4 to 1.7 ↓ Visual analog scale from 9 to 5; improvements to invalidating behavior problems including screaming and aggression Side effects: mouth ulcers when tincture was used***	Broers et al., 2019^

Detrimental effects of the treatment are indicated by “***”. “^” denotes where clinical trial registration numbers were not reported in the study and could not be located retrospectively on clinical trial registries.

Aβ, Amyloid-β; AD, Alzheimer’s disease; *APP/PS1*, *APP_Swe_/PS1*Δ*E9* mouse model; BPSD, behavioral and psychological symptoms of dementia; CB1R, cannabinoid 1 receptor; CBD, cannabidiol; CBN, cannabinol; ECS, endocannabinoid system; H_2_O_2_, hydrogen peroxide; IC_50_, half-maximal inhibitory concentration; i.p., intraperitoneal; p.o., per os; ROS, reactive oxygen species; s.c., subcutaneous; THC, delta-9-tetrahydrocannabinol; WT, wild type-like.

*In vitro*, co-administration of CBD and THC resulted in an additive neuroprotection of mouse hippocampal nerve cells (HT22 cells) against glutamate toxicity, while co-administration with both THC and CBN resulted in synergistic neuroprotection that was greater than a purely additive effect of the individual isolates (Schubert et al., 2019). These findings highlight the synergistic fashion in which cannabinoids can act to exert therapeutic effects. Interestingly, RNA sequencing suggested other mechanisms beyond the ECS as being relevant for the observed beneficial effects (Schubert et al., 2019). Another study in SH-SY5Y (human neuroblastoma) neuronal cells found that pure THC had higher antioxidant potency than pure CBD, and accordingly, that the lowest THC-containing combination (90:10 CBD:THC) was the least effective at decreasing ROS level (Raja et al., 2020). Conversely, other ratios with high fractions of CBD (e.g., 25:75 CBD:THC) possessed increased antioxidant properties compared to treatments with lower CBD fractions (e.g., 10:90 CBD:THC), suggesting complex ratio-specific interactions between THC and CBD. Of the cannabis extracts assessed and in line with the cannabinoid isolate findings, THC-rich (∼72% THC, no detectable CBD) cannabis extracts showed the greatest antioxidant activity, whereas CBD-rich cannabis extracts (containing 50.3–64.3% CBD and 3.9–11.5% THC) were less effective at reducing ROS (Raja et al., 2020). These studies suggest complex, and, to a degree, synergistic relationships between cannabinoids, which affect their antioxidant properties (see Figure 1) and demand a cautious approach (both in the selection of particular cannabinoids, their ratios, and the potential use of extracts) when determining the optimal multi-cannabinoid treatment strategy to achieve positive therapeutic effects for AD. Nonetheless, combinations of THC and CBD (in purified forms or as cannabis extracts) appear to have promising antioxidant potential for AD *in vitro*.

Importantly, *in vivo* studies confirm that cannabis extracts might be beneficial for dementia therapy. Chronic treatment of 6-month-old male *APP/PS1* mice with either a CBD-rich (64.8% CBD, 2.3% THC), THC-rich (67.1% THC, 0.3% CBD), or 1:1 CBD:THC combination extract (all 0.75 mg/kg for each cannabinoid) reversed object recognition memory deficits of AD transgenic mice. However, only *APP/PS1* mice treated with the 1:1 CBD:THC combination extract recovered from a learning impairment in the active avoidance test as well (Aso et al., 2015). Furthermore, while all treatments reduced the number of astrocytes found around Aβ plaques, only 1:1 CBD:THC combination extract reduced levels of soluble cortical Aβ_42_, microgliosis and neuroinflammation, and provided protection from oxidative damage *via* increased protein levels of thioredoxin 2 (a key component of mitochondrial antioxidant systems). Thus, chronic administration of combined 1:1 CBD:THC cannabis extract during the early symptomatic stage possessed superior therapeutic potential than administration of CBD-rich or THC-rich extracts. In a later study in 12-month-old *APP/PS1* mice (i.e., advanced stages of AD), 1:1 CBD:THC combination extract was still effective in reversing the memory deficit in the two-object recognition task of these older AD mice (no effects on non-AD transgenic control mice), but unable to alter amyloid pathology or gliosis at this later age (Aso et al., 2016b). These studies highlight that 1:1 CBD:THC combination extracts appear more protective than either a THC- or CBD-rich extract in this mouse model system, and importantly may be beneficial for treating AD in early disease stages. See Figure 1 for a summary of CBD+THC effects in AD.

Further evidence from other non-AD (but related) *in vivo* models confirms insights provided by Aso and colleagues. A study in a complex tauopathy mouse model (PK^–/–^/Tau^VLW^) resembling a multisystemic neurological disease (e.g., frontotemporal dementia with parkinsonism and amyotrophy) found that chronic treatment with 1:1 CBD:THC cannabis extract combination (1.5 mg/kg each CBD and THC) was beneficial against several AD-related pathologies including neuroinflammation, gliosis, and the deposition of both Aβ and tau in the hippocampus and cerebral cortex (see Figure 1) and also reduced aggressive behaviors of compared to vehicle-treated PK^–/–^/Tau^VLW^ mice (Casarejos et al., 2013). In a mouse model of Lafora disease, CBD-rich cannabis extract (35 mg/kg CBD, 4.8 mg/kg THC, plus other minor cannabinoids) reversed cognitive impairments of 12-month-old malin knock-out mice (Aso et al., 2020). Further, CBD-rich cannabis extract-supplemented diet (38.8% CBD in standard diet) increased hippocampal neurogenesis in 16-week-old female C57BL/6 mice although spatial memory was not improved compared to control diet-fed animals (Wolf et al., 2010). Importantly, neurogenesis is severely impaired in AD (Mu and Gage, 2011; Babcock et al., 2021), and recent studies suggest that stimulation of neurogenesis may be a viable therapeutic pathway for AD (Babcock et al., 2021).

Use of cannabis combination therapies for AD in *in vivo* studies is complicated by the fact that cannabis, and in particular, THC can be detrimental to cognition. However, in the context of multi-cannabinoid therapy strategies, it is relevant to note that whether detrimental effects are seen appears to be dependent on age and the amount of THC co-administered. For example, the pro-neurogenic effect of CBD-rich cannabis extract seen in C57BL/6 mice (Wolf et al., 2010) was not evident in THC-rich diet-fed (41.2% THC in standard diet) animals and those mice also exhibited reduced spatial learning and memory (Wolf et al., 2010). Furthermore, THC-rich extract treatment (0.75 mg/kg) of control mice resulted in reduced recognition index in the two-object recognition task when compared to vehicle-treated control mice (Aso et al., 2015). However, THC-induced impairments of cognition appeared only evident in young and healthy animals, and important to AD-therapy, low-dose THC and low-dose 1:1 CBD:THC had no cognitive-impairing properties in older or AD transgenic animals and instead improved cognition (as had been outlined above). Indeed, the 1:1 CBD:THC-rich cannabis extract treatment used by Aso and colleagues did not have a detrimental effect on control mice, indicating that the addition of CBD prevented the damaging effects of THC to memory (Aso et al., 2015). Thus, the current evidence suggests that multi-cannabinoid treatment strategies using combinations with low-dose THC or administration of THC-low cannabis extracts (or indeed 1:1 CBD:THC-rich cannabis extracts) might be the best option for AD therapy. However, this potential approach needs further evaluation as for example, 1:1 CBD:THC (1 mg/kg each) did not affect spatial learning of 18-month-old male mice despite treatment with 1 mg/kg THC alone restored age-related cognitive decline (Nidadavolu et al., 2021). Furthermore, 2 months of treatment with CBD-rich cannabis extract (35 mg/kg CBD, 4.8 mg/kg THC) was detrimental to the recognition index of 6-month-old control (WT) mice (Aso et al., 2020).

The insights gained from the currently available *in vitro* and *in vivo* data, multi-cannabinoid treatment strategies show potential as a future therapeutic option for AD. The evidence available to date highlights that commencing treatment in the early symptomatic phase, with multi-cannabinoid formulas low in THC (or at a 1:1 CBD:THC ratio) may result in the best therapeutic outcome for AD while also reducing the chance of detrimental effects. Importantly, further research is required including clinical studies for multi-cannabinoid combination therapy in AD. Current clinical trials testing multi-cannabinoid treatment strategies are mostly ongoing or planning to commence (e.g., NCT04075435, NCT03328676, NCT05239390, ACTRN12619000474156, and 2020-001056-17). Early evidence suggests that combination therapy may be beneficial in decreasing neuropsychiatric symptoms, some behavioral impairments, and agitation. A 2016 prospective open label trial in 10 people with AD and BPSD found that 4 weeks of a titrated dosage of THC-rich cannabis extract (from 2.5 to 7.5 mg THC given orally twice per day) reduced neuropsychiatric symptoms including improvements to agitation, irritability, sleep, apathy, and delusions (Shelef et al., 2016). The study also found that treatment reduced the severity of the overall Clinical Global Impression. However, confusion was noted as a side effect in one person at 5 mg THC. Similarly, a 2019 prospective observational pilot study in 10 women with severe dementia and BPSD found that 2 months of a titrated dosage of a CBD:THC tincture or oil (THC up to 9 mg/day, CBD twice that of THC) reduced neuropsychiatric symptoms, agitation, and rigidity and resulted in improvements to invalidating behaviors such as screaming and aggression (Broers et al., 2019). Improvements in daily care and other domains were observed by the nurses. Importantly, several study participants were able to reduce or stop opioid therapies (for pain) which resulted in ceased constipation, and others stopped or reduced their use of antipsychotic and anti-anxiety medication. A side effect noted in this study was that the formation of mouth ulcers with the use of the tincture form of the medication. Unfortunately, small sample sizes of the above studies and lack of appropriate control groups mean that these results should be treated with caution, however, the findings do point toward the acceptability and tolerability of cannabinoid combination therapies for AD in humans (Shelef et al., 2016; Broers et al., 2019). Randomized, double-blind, placebo-controlled clinical trials are needed for further evaluation of the use of multi-cannabinoid treatment strategies for not only behavioral and neuropsychiatric symptoms of dementia, but more importantly, cognitive decline and pathological hallmarks of AD.

## Clinical and legal considerations of cannabis use in Alzheimer’s disease

Access to cannabis-based medicines is tightly regulated in Australia and subject to approval by the Therapeutics Goods Administration (TGA), ensuring standardized, high-quality products. In Australia, CBD preparations containing a maximum of 2% other naturally derived cannabinoids (including THC) is a Schedule 4 Prescription Only Medicine, with preparations of 150 mg or less CBD with no more than 1% THC being classed as Schedule 3 Pharmacist Only Medicines (i.e., over-the-counter). THC, THC-containing cannabis extracts, and nabiximols (i.e., Sativex 1:1 CBD:THC extract oromucosal spray) are classed as Schedule 8 Controlled Drugs. Nabiximols and epidyolex (CBD) are the only two cannabis products currently available on the Australian Register of Therapeutic Goods (ARTG); all other unregistered medicinal cannabis products can be accessed through the Special Access Schemes and Authorized Prescriber pathways. There is currently no cannabis product registered specifically for use in AD. Internationally, the situation regarding the legalization and decriminalization of medicinal cannabis varies markedly. For example, in Canada, both medicinal and recreational cannabis is legal at the federal level, and in the Netherlands cannabis use is “tolerated,” and legal as medicinal cannabis. In the United States, cannabis (excluding CBD derived from hemp) is illegal at the federal level, although there are certain states that have medical cannabis programs.

Consideration of formulation, administration method, and dosage on a case-by-case basis will be important in the prescription of medicinal cannabis products for individuals with AD. THC-rich formulations may be more relevant for use in managing BPSD in people living with advanced AD, while CBD-rich preparations could have more potential when used in a prophylactic setting for preclinical, prodromal (i.e., mild cognitive impairment) or mild AD (Steiner-Lim et al., under review). Use of low-THC treatment regimes in younger onset AD (onset < 65 years) would also be preferable given the potential side effects of THC-rich formulations (e.g., fatigue, disorientation) in younger people, whom may operate heavy machinery, drive, be employed, or have dependent children.

Driving post consumption of cannabis-based medicines containing THC presents a problem to those considering use of cannabis products, given the impairing effects of THC and legal repercussions (for more details, see Arkell et al., 2019). For example, detection of THC during roadside mobile drug testing in Australia can result in prosecution, while CBD, which alone does not appear to impair driving performance (McCartney et al., 2022), is not monitored. Currently under consideration are changes to Victorian legislation to allow users of THC-containing medicinal cannabis to legally drive, providing they hold a lawful prescription and are not impaired while driving (determined for example by a roadside impairment test or by a certificate of fitness from a general practitioner) (Arkell et al., 2021).

## Conclusion

This mini review highlights that multi-cannabinoid combination treatment strategies are valid candidates for novel AD therapies. However, further investigations, and in particular, clinical studies, are required to determine optimal dose and ratio of cannabinoids for treatment of behavioral, cognitive, and pathological symptoms of AD thereby also considering other cannabinoids in addition to the current focus on THC and CBD (most cannabis extract studies did not profile cannabinoid content beyond those two phytocannabinoids). Importantly, all relevant studies reviewed were carried out in one sex/gender only. Given that sex-specificity is evident in AD transgenic mouse models (van Eersel et al., 2015; Jiao et al., 2016) and gender differences are seen in dementia (Oveisgharan et al., 2018) as well as in the ECS and the response to cannabis (Craft et al., 2013), it is pertinent that these future investigations consider both sexes.

## Author contributions

MC, GZS, and TK involved in writing the manuscript. All authors contributed to the article and approved the submitted version.
